# Patient-reported outcomes in a pilot clinical trial of twice-weekly hemodialysis start with adjuvant pharmacotherapy and transition to thrice-weekly hemodialysis vs conventional hemodialysis

**DOI:** 10.1186/s12882-022-02946-w

**Published:** 2022-09-27

**Authors:** Mariana Murea, Benjamin R. Highland, Wesley Yang, Emily Dressler, Gregory B. Russell

**Affiliations:** 1 Department of Internal Medicine, Section on Nephrology, Wake Forest School of Medicine, Medical Center Boulevard, Winston-Salem, NC 27157-1053 USA; 2 Department of Internal Medicine, Wake Forest School of Medicine, Winston-Salem, NC USA; 3 Department of Biostatistics and Data Science, Division of Public Health Sciences, Wake Forest School of Medicine, Winston-Salem, NC USA

**Keywords:** Incremental, Hemodialysis, Patient-reported outcomes

## Abstract

**Background:**

Physical and emotional symptoms are prevalent in patients with kidney-dysfunction requiring dialysis (KDRD) and the rigors of thrice-weekly hemodialysis (HD) may contribute to deteriorated health-related quality of life. Less intensive HD schedules might be associated with lower symptom and/or emotional burden.

**Methods:**

The TWOPLUS Pilot study was an individually-randomized trial conducted at 14 dialysis units, with the primary goal to assess feasibility and safety. Patients with incident KDRD and residual kidney function were assigned to incremental HD start (twice-weekly HD for 6 weeks followed by thrice-weekly HD) vs conventional HD (thrice-weekly HD). In exploratory analyses, we compared the two treatment groups with respect to three patient-reported outcomes measures. We analyzed the change from baseline in the score on Dialysis Symptom Index (DSI, range 0–150), Generalized Anxiety Disorder-7 (GAD-7, range 0–21), and Patient Health Questionnaire-9 (PHQ-9, range 0–27) at 6 (*n* = 20 in each treatment group) and 12 weeks (*n* = 21); with lower scores denoting lower symptom burden. Analyses were adjusted for age, race, gender, baseline urine volume, diabetes mellitus, and malignancy. Participants’ views on the intervention were sought using a Patient Feedback Questionnaire (*n* = 14 in incremental and *n* = 15 in conventional group).

**Results:**

The change from baseline to week 6 in estimated mean score (standard error; *P* value) in the incremental and conventional group was − 9.7 (4.8; *P* = 0.05) and − 13.8 (5.0; *P* = 0.009) for DSI; − 1.9 (1.0; *P* = 0.07) and − 1.5 (1.4; *P* = 0.31) for GAD-7; and − 2.5 (1.1; *P* = 0.03) and − 3.5 (1.5; *P* = 0.02) for PHQ-9, respectively. Corresponding changes from week 6 to week 12 were − 3.1 (3.2; *P* = 0.34) and − 2.4 (5.5; *P* = 0.67) in DSI score; 0.5 (0.6; *P* = 0.46) and 0.1 (0.6; *P* = 0.87) in GAD-7 score; and − 0.3 (0.6; *P* = 0.70) and − 0.5 (0.6; *P* = 0.47) in PHQ-9 score, respectively. Majority of respondents felt their healthcare was not jeopardized and expressed their motivation for study participation was to help advance the care of patients with KDRD.

**Conclusions:**

This study suggests a possible mitigating effect of twice-weekly HD start on symptoms of anxiety and depression at transition from pre-dialysis to KDRD. Larger clinical trials are required to rigorously test clinically-matched incrementally-prescribed HD across diverse organizations and patient populations.

**Trial registration:**

Registered at ClinicalTrials.gov with study identifier NCT03740048, registration date 14/11/2018.

**Supplementary Information:**

The online version contains supplementary material available at 10.1186/s12882-022-02946-w.

## Introduction

Dialysis initiation is a stressful experience for patients, with the initial months of treatment being critical in terms of both adaptation and mortality [1–4]. Thus, it is not surprising that health-related quality of life in patients with kidney dysfunction requiring dialysis (KDRD) is substantially lower than the general population, a finding consistent across three continents in one analysis from the Dialysis Outcomes and Practice Patterns Study (DOPPS) [5]. Symptoms of anxiety and/or depression are present in 25–50% of patients initiating hemodialysis (HD) [6]. In a cross-sectional study involving patients with prevalent KDRD on maintenance HD, Weisbord et al. used the Dialysis Symptom Index (DSI) instrument and showed that 79% of the patients had symptoms of dry skin, 69% had fatigue, 50% had bone or joint pain, 49% had dry mouth, 45% had insomnia, 20% had feelings of sadness, and 31% had feelings of anxiety [7]. However, few studies examined longitudinal patient-reported psychosomatic symptomatology after HD initiation and little is known about processes of care that impact psychological burden of HD [8]. A few recent studies in patients with KDRD showed that patient-reported symptom burden, emotional well-being, and spiritual well-being vary widely from month to month regardless of whether overall trajectories improve or worsen over time [9–11]. Therefore, serial collection of patient-reported health-related quality of life is a requisite for an accurate depiction of a full spectrum and dynamic of physical and emotional symptoms.

Dialysis initiation provokes substantial changes in lifestyle that require significant adjustments due to the intrusiveness of disease treatment into multiple life domains, which may underlie the greater than expected rates of anxiety [12]. Consequently, it has been hypothesized that personalized HD therapy consisting of less frequent HD treatments at dialysis initiation in those who have suitable levels of residual kidney function, with subsequent adaptations in dialysis frequency to thrice-weekly or more often HD as residual kidney function declines—i.e., incremental HD—could be conducive to better adjustment to life style changes and better health-related quality of life. In spite of the alluring attributes of incremental HD rendered by other potential benefits such as better or non-inferior patient survival for significant economic savings, high-quality research focused on clinical outcomes and patient-reported outcomes with incremental HD is scarce [13, 14]. Registry-based studies showed that about 30% of patients with incident KDRD could be treated with an initial schedule of less frequent HD based on their residual kidney function and morbidity profile [15], yet incremental HD remains grossly underused in the United States [16], possibly due to lack of clinical trials to validate the safety of this treatment approach as well as other potential barriers reviewed in former publications [17].

Current data on incremental HD and patient outcomes is based on observational, retrospective studies and the results are mixed. In one study done in China, quality of life scores did not differ between the twice-weekly and thrice-weekly HD groups [18]. Other studies, however, surmised that incremental-start HD, by virtue of better preservation of residual kidney function, confers better quality of life [19, 20]. Thus, comparative effectiveness research in randomized clinical trials needs to be performed to elucidate, among other outcomes, longitudinal differences in psychosomatic symptomatology between incremental HD and conventional HD.

We undertook the first pilot clinical trial of incremental-start HD in patients with incident KDRD in the US (NCT 03740048). The primary objective of the TWOPLUS pilot clinical trial was to test the feasibility of implementing a schedule of incremental HD at outpatient dialysis facilities using a protocol of blood tests and timed urine collection that was embedded in usual clinical workflow, the results of which were published elsewhere [21]. Exploratory analyses compared patient-reported outcomes using three instruments, the results of which are presented here. Using semi-structured interviews, we also evaluated participant’s perceptions on the intervention and provider perceived barriers to incremental HD.

## Methods

### Trial design and oversight

We conducted this prospective, individually-randomized, unblinded controlled clinical trial at 14 outpatient dialysis facilities in North Carolina, US [22]. The trial protocol was approved by the relevant health authorities and the Institutional Review Board of Wake Forest School of Medicine in North Carolina, USA; and was registered with the Clinical Trials Registry (NCT03740048). This was an academic investigator–initiated trial, funded by Relypsa, Vifor Pharma (IE19–00819/GTS47902). All the patients provided written informed consent and fulfilled all eligibility criteria before randomized treatment allocation. The study was carried out in accordance with Good Clinical Practice guidelines and the Declaration of Helsinki.

### Eligibility criteria

These are summarized in Table S1. Excluded were patients with eGFR ≥5 mL/min/1.73m^2^ and urine output of < 500 mL/day at the time of HD initiation, and abrupt decline in kidney function as result of severe acute kidney injury (stage 3 AKI defined by Acute Kidney Injury Network [AKIN]) preceding HD initiation.

### Randomization and treatments

Patient recruitment began on June 14, 2019, was paused between March 13, 2020 and May 31, 2020 due to COVID-19 pandemic, and resumed on June 01, 2020. The study enrolled adults patients with incident KDRD who had sufficient residual kidney function at dialysis initiation, characterized by presence of urine volume of ≥500 mL/day and estimated glomerular filtration rate of ≥5 mL/min/1.73 m^2^. A detailed study protocol along with full eligibility criteria has been published [22]. Participant recruitment occurred at Wake Forest Outpatient Nephrology Clinics, Wake Forest Inpatient Nephrology Service, and Wake Forest Outpatient Dialysis facilities. Randomization was determined by a computer algorithm, in random blocks of 2 or 4 size and 1:1 allocation, stratified by type of vascular access used at HD initiation (catheter or arteriovenous access). Participants were assigned to one of the two HD regimens: (1) twice-weekly HD and clinically-indicated adjunctive pharmacological therapy (loop diuretic, potassium-binding agent (patiromer) and sodium bicarbonate) for six consecutive weeks, continued by thrice-weekly HD (incremental HD group); or (2) thrice-weekly HD (conventional HD group). Patients were recruited by a member of the study team (e.g., physician investigator, study coordinator) and the allocation sequence was generated automatically by a computerized system.

### Study procedures

For practicality and in the absence of data regarding optimal level of urea solute clearance in patients with incident KDRD and residual kidney function, the dialysis prescription was adjusted to achieve dialysis single-pool Kt/Vurea (spKdt/Vurea) of ≥1.2 and urea reduction ration (URR) of ≥65% in both treatment groups throughout the study period—including during the first 6 weeks in the incremental HD group. Under the direction of the treating nephrologist, progression from twice-weekly to thrice-weekly HD was allowed to take place prior to the six-week time point for clinical manifestations (e.g., uncontrolled uremic symptoms, volume overload, persistent biochemical imbalances) deemed to benefit from more frequent HD. Adjuvant pharmacological therapy (ie, loop diuretics± thiazide diuretics, patiromer potassium-binding agent and/or bicarbonate-based agent) were prescribed during the period of twice-weekly HD in the incremental HD group, as previously detailed [22].

### Outcomes and measurements

Pre-specified exploratory analyses, reported here, included comparison in longitudinal changes in patient-reported outcomes using three questionnaires; and collection of participant feedback on study-related procedures using a semi-structured questionnaire. Sample size selection for the primary outcome of feasibility of incremental HD has been previously described [22]. Questionnaire-based patients’ answers were gathered through telephone interview by a study team member (i.e., study coordinator) trained in questionnaire administration. Data was logged as missing when participants declined interview participation.

#### Survey instruments

##### Symptoms

To assess physical and emotional symptoms and their severity, we used the Dialysis Symptom Index (DSI), an instrument shown to have been validated in the dialysis population [7, 8]. The DSI contains 30 items, each of which targets a specific physical or emotional symptom. Participants were asked to report the presence (yes/no) of each symptom at any time during the previous 7 days. The instrument uses a five-point Likert scale (1, “not at all bothersome”, to 5, “bothers very much”) to rate the severity of each symptom reported as being present. To generate an overall symptom severity score for each patient, we summed the individual severity scores for all of the symptoms that were reported on the DSI. Symptoms that were not reported as being present were assigned a severity score of zero. Thus, the minimum possible overall severity score was zero when none of the 30 symptoms were reported, and the maximum potential overall severity score was 150 when all 30 symptoms were reported and rated as “bothers very much” (Likert-scale severity score of 5). The questions referred to the past week prior to the time point of questionnaire administration.

##### Anxiety

Symptoms of anxiety were assessed with the 7-item Generalized Anxiety Disorder (GAD-7) instrument. Responses for each item are rated on a scale of 0–3 (0 “not at all bothered” and 3 “bothered nearly every day”). A sum score ranging between 0 and 21 indicates severity, with higher scores representing more severe anxiety [23]. The questions referred to the past 2 weeks prior to the time point of questionnaire administration.

##### Depression

Depressive symptoms were evaluated using the 9-item Patient Health Questionnaire (PHQ-9), which is a reliable scale in the dialysis population that has been validated previously by comparing it with the gold standard (i.e., the clinical interview) [24, 25]. The PHQ-9 evaluates symptoms of depression experienced by the respondent over the last 2 weeks. It is a comprehensive depression screening tool, as it covers all the 9 criteria, from the diagnostic and statistical manual of mental disorders, 4th edition (DSM-IV) for diagnosis of major depressive disorders. The PHQ-9 form comprises four Likert scale responses, akin to the GAD-7 instrument. In total, depression scores can range from 0 to 27 with higher scores representing more severe symptoms [26]. The questions referred to the past 2 weeks prior to the time point of questionnaire administration.

##### Participant feedback

We devised a 24-item Patient Feedback Questionnaire to assess 5 domains (information and communication, coordination of care, perception on study-related assessments, motivation, and future studies) in patients participating in the TWOPLUS Pilot clinical trial (Table S2). Answers were rated on a 5-level scale to (1) evaluate the delivery of study-related information, (2) evaluate coordination of care during the study as it pertained to study-related procedures, (3) evaluate patient perceptions on study-related assessments, and (4) elicit patients’ motivations for study participation.

### Study follow-up and data collection

Baseline demographic characteristics were obtained at study enrollment. All study visits occurred during the patients’ regularly scheduled HD session at Wake Forest Outpatient Dialysis facilities. Questionnaires were administered at baseline, 6 weeks and 12 weeks, prior to dialysis initiation on a dialysis day. The semi-structure questionnaires to obtain participant feedback was administered between 24 and 48 weeks via telephone interview.

### Statistical analysis

Continuous variables are summarized with mean (95% confidence interval [CI]) and categorical variables are given as proportion per participant or per visit as appropriate. Changes in DSI, GAD-7 and PHQ-9, tested at successive time points, were analyzed between treatment groups at each assessment time point (week 6, week 12, and week 24) using pairwise comparisons with a repeated measures (longitudinal) mixed effect regression model; these models provided estimated changes in least squares mean and corresponding standard errors. Model 1 represents unadjusted analyses; Model 2 includes adjustments for covariates of age, race, gender, baseline urine volume, diabetes mellitus status, and malignancy status. As these analyses were exploratory in nature, all analyses and pairwise comparisons were conducted with significance assumed if the observed *P* value was < 0.05. There were no adjustments for multiple comparisons. The analysis was conducted by original assigned groups. Statistical analyses were performed using SAS, version 9.4, Cary, NC, USA.

## Results

### Participant characteristics

**Fig. 1:**
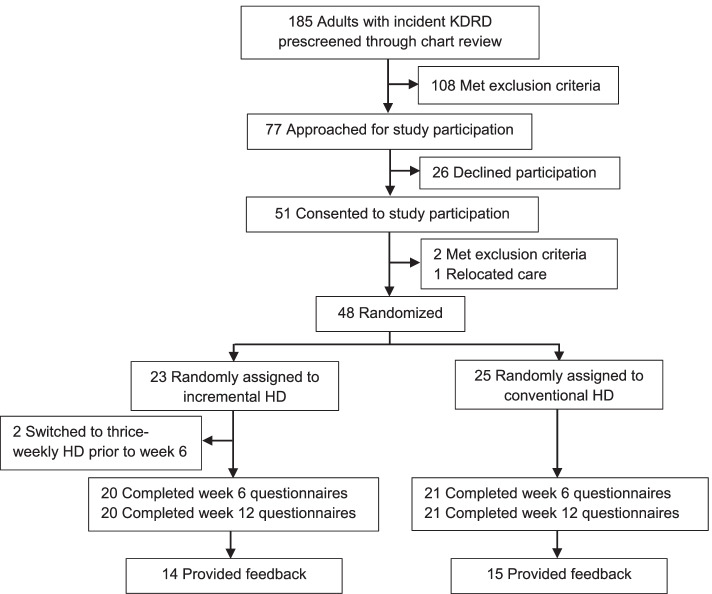
Participant flow through the study. KDRD denotes dialysis-dependent kidney disease; HD, hemodialysis

Of 185 patients with new-onset KDRD who were started on HD during the enrollment period, 77 (42%) met preliminary eligibility criteria, 51 (28%) consented to study participation, and 48 (26%) were included in the trial (Fig. 1). All participants had received between 3 and 5 HD sessions prescribed thrice-weekly by the treating nephrologist, before randomization. Baseline demographic and clinical data for the two groups have been described in a separate report [21]. Briefly, the cohort had mean (SD) age 61.3 (14.0) years, 44% were women, 56% were Black, 65% had diabetes and 79% were using a central venous catheter at enrollment (Table 1). Participants were followed for 12 months or until a drop-out event, totaling to a mean follow-up duration of 288.9 days in the incremental-start group and 275.3 days in the conventional group [21].

**Table 1:** Characteristics of the participants at enrollment

Characteristics	Overall (***n*** = 48)	Incremental HD (***n*** = 23)	Conventional HD (***n*** = 25)
Age at enrollment (years), mean (SD)	62.02 (14.74)	59.61 (15.33)	64.33 (13.76)
Female, n (%)	21 (43)	10 (43)	11 (42)
Race, n (%)			
White	19 (40)	11 (48)	8 (33)
Black	26 (55)	11 (48)	16 (63)
Hispanic	2 (4)	1 (4)	1 (4)
KDRD etiology, n (%)			
Diabetes mellitus	24 (49)	9 (39)	15 (58)
Glomerulonephritis/Vasculitis	2 (4)	2 (9)	0 (0)
Other/Unknown	23 (47)	12 (52)	11 (42)
Vascular Access^a^, n (%)			
Arteriovenous fistula	9 (19)	5 (22)	4 (17)
Arteriovenous graft	1 (2)	0 (0)	1 (4)
Central venous catheter	38 (79)	18 (78)	20 (79)
Comorbidities, n (%)			
Diabetes mellitus	30 (63)	12 (52)	18 (72)
Coronary artery disease	8 (17)	4 (17)	4 (16)
Congestive heart failure	11 (23)	4 (17)	7 (28)
Peripheral arterial disease	5 (10)	2 (9)	3 (12)
Cerebrovascular disease	8 (17)	3 (13)	5 (20)
Malignancy	9 (19)	3 (13)	6 (24)
HIV	2 (4)	2 (9)	0 (0)
Anxiety	2 (4)	0 (0)	2 (8)
Depression	3 (6)	2 (9)	1 (4)
Drug abuse	1 (2)	0 (0)	1 (4)
Alcohol abuse	2 (4)	2 (9)	0 (0)
Medications			
Anxiolytics	1 (2)	0 (0)	1 (4)
Antidepressants	0 (0)	0 (0)	0 (0)
HD prescription^b^, mean (SD)			
Treatment time (minutes)	201.06 (35.09)	204.13 (38.75)	198.13 (30.92)
Blood flow (mL/min)	304.26 (60.87)	295.65 (62.40)	312.50 (58.18)
Dialysate flow (mL/min)	540.43 (84.20)	530.43 (74.80)	550.00 (91.29)

^a^Vascular access used at dialysis initiation

^b^HD prescription parameters represent the last HD, prescribed thrice-weekly by treating provider prior to study enrollment and randomization

Percentages may not total 100 because of rounding. *KDRD* denotes kidney dysfunction requiring dialysis, *HD* hemodialysis, *SD* standard deviation

### Patient-reported outcomes

All participants completed the questionnaires at baseline. Over the duration of the study and after excluding those participants who died or were lost to follow-up, the rate of completion of scheduled questionnaires was 87% (20 of 23) in the incremental and 84% (21 of 25) in the conventional HD group; and the rate of participant feedback was 61% (14 of 23) and 60% (15 of 25), respectively. Baseline mean (95% CI) scores obtained pre-randomization were similar for DSI and GAD-7 in both treatment groups, and lower for PHQ-9 in incremental group (Table 2).

**Table 2:** Longitudinal changes in patient-reported outcomes, residual kidney function, and HD treatment time

Assessment	Baseline				Week 6				Week 12			
	Incremental HD		Conventional HD		Incremental HD		Conventional HD		Incremental HD		Conventional HD	
	N	Mean (95% CI)	N	Mean (95% CI)	N	Mean (95% CI)	N	Mean (95% CI)	N	Mean (95% CI)	N	Mean (95% CI)
*Model 1*												
**DSI**	20	39.5 (26.6, 52.4)	21	41.5 (28.2, 55.7)	20	29.2 (20.1, 38.2)	21	27.5 (18.5, 36.6)	20	25.8 (18.8, 32.9)	21	24.8 (17.6, 32.0)
**GAD-7**	20	3.5 (1.0, 6.0)	20	3.5 (1.0, 6.6)	20	1.6 (0, 3.4)	20	3.4 (1.5, 5.2)	20	2.0 (0.6, 3.4)	20	2.9 (1.5, 4.2)
**PHQ-9**	20	5.5 (2.7, 8.3)	20	6.7 (3.8, 9.5)	20	3.0 (1.5, 4.5)	20	3.9 (2.4, 5.4)	20	3.3 (1.9, 4.6)	20	3.3 (2.0, 4.6)
**Urine volume, mL/24 h**	23	914 (654–1174)	25	1424 (976–1872)	19	504 (335–673)	17	553 (384–723)	19	479 (288–670)	19	484 (292–676)
**Renal urea clearance, mL/min/1.73 m**^**2**^	18	3.3 (2.0–4.5)	17	4.1 (2.9–5.3)	19	2.3 (1.6–2.9)	17	2.3 (1.6–3.0)	19	2.1 (1.4–2.8)	19	2.0 (1.4–2.7)
**HD treatment time (min)**	23	232.6 (24.8)	25	209.2 (21.9)	21	234.8 (27.3)	21	214.8 (34.3)	20	230.6 (23.5)	20	211.4 (29.8)
**Dialysis spKt/V**^a^	22	1.43 (1.32–1.53)	21	1.30 (1.20–1.40)	22	1.42 (1.32–1.51)	18	1.40 (1.30–1.50)	21	1.42 (1.34–1.50)	18	1.36 (1.29–1.45)
*Model 2*												
**DSI**	20	42.6 (29.5, 55.8)	21	41.3 (27.5, 55.0)	20	33.0 (23.4, 42.6)	21	27.5 (17.3, 37.7)	20	29.9 (21.2, 38.6)	21	25.9 (16.6, 35.2)
**GAD-7**	20	3.6 (1.1, 6.2)	20	3.1 (0.5, 5.8)	20	1.7 (0, 3.6)	20	3.0 (1.0, 5.0)	20	2.1 (0.4, 3.9)	20	3.1 (1.3, 4.9)
**PHQ-9**	20	5.6 (2.9, 8.4)	20	6.7 (3.8, 9.5)	20	3.1 (1.5, 4.8)	20	4.1 (2.4, 5.9)	20	3.4 (1.8, 5.0)	20	3.7 (1.9, 5.4)

Model 1 represents unadjusted analyses; Model 2 includes adjustments for covariates of age, race, gender, baseline urine volume, diabetes mellitus status, and malignancy status. *DSI* denotes Dialysis Symptom Index, *GAD-7* Generalized Anxiety Disorder 7-item, *HD* Hemodialysis, *LS* Least squares, *PHQ-9* Patient Health Questionnaire 9-item, *SE* Standard error

^a^spKt/V, single pool Kt/V urea per HD session as reported according to usual care and monthly lab testing at outpatient dialysis units, without adjustment for HD sessions per week

**Fig. 2:**
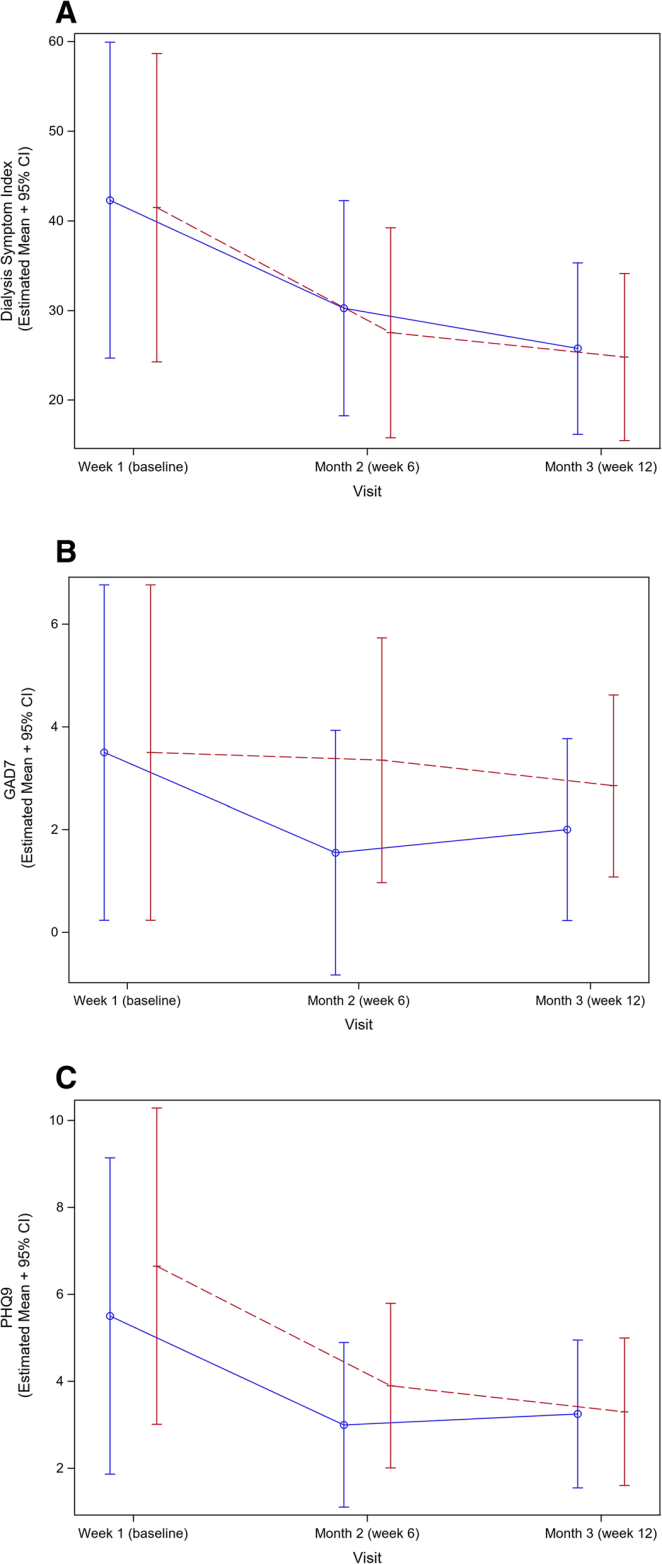
Patient reported outcomes over time using (**A**) Dialysis Symptoms Index (DSI), (**B**) Generalized Anxiety Disorder 7 items (GAD-7), and (**C**) Patient Health Questionnaire 9 items (PHQ-9) instrument. Trajectories are presented in blue line for incremental HD group, and red line for conventional HD group

The DSI score decreased in both treatment groups at week 6 and continued to decrease by week 12 (Fig. 2A). After adjustment for baseline covariate differences, the least square mean change (standard error [SE]) in DSI was borderline significant in the incremental group (− 9.7 [4.8], *P* = 0.05) and it remained strongly significant in the conventional group (− 13.8 [5.0], *P* = 0.009). Between week 6 and 12, the change in DSI was not statistically significant in either treatment group (Table 3).

**Table 3:** Least squares mean change in patient-reported outcomes within each group

Study Period	Baseline to Week 6				Week 6 to Week 12			
	Incremental HD		Conventional HD		Incremental HD		Conventional HD	
	LS mean change (SE)	*P* value	LS mean change (SE)	*P* value	LS mean change (SE)	*P* value	LS mean change (SE)	*P* value
*Model 1*								
**DSI**	−10.3 (4.7)	0.03	−14.0 (4.8)	0.005	−3.3 (3.2)	0.30	−2.7 (3.2)	0.40
**GAD-7**	−2.0 (1.0)	0.03	−0.2 (1.0)	0.89	0.5 (0.7)	0.25	−0.5 (0.7)	0.60
**PHQ-9**	−2.5 (1.1)	0.04	−2.8 (1.1)	0.01	0.3 (0.6)	0.70	−0.6 (0.6)	0.34
*Model 2*								
**DSI**	−9.7 (4.8)	0.05	−13.8 (5.0)	0.009	−3.1 (3.2)	0.34	−2.4 (5.5)	0.67
**GAD-7**	−1.9 (1.0)	0.07	−1.5 (1.4)	0.31	0.5 (0.6)	0.46	0.1 (0.6)	0.87
**PHQ-9**	−2.5 (1.1)	0.03	−3.5 (1.5)	0.02	−0.3 (0.6)	0.70	−0.5 (0.6)	0.47

Model 1 represents unadjusted analyses; Model 2 includes adjustments for covariates of age, race, gender, baseline urine volume, diabetes mellitus status, and malignancy status. *DSI* denotes Dialysis Symptom Index, *GAD-7* Generalized Anxiety Disorder 7-item, *LS* least squares, *PHQ-9* Patient Health Questionnaire 9-item, *SE* standard error

The GAD-7 decreased to a larger extent in incremental group during the first 6-week interval, in contrast to a slight change in score in the conventional group (Fig. 2B and Table 3). By week 12, the GAD-7 score rose in incremental group, while there was a slight decline in conventional group (Fig. 2B). Multivariate adjustment reduced the strength of the association between the least square mean change in GAD-7 and incremental HD (− 1.9 [1.0], *P* = 0.07) that was seen with unadjusted analyses (− 2.0 [1.0], *P* = 0.03) in the first 6 weeks of dialysis. There was no association between conventional HD and change in GAD-7, and after the first 6 weeks of dialysis there was no association between incremental HD and GAD-7 scores (Table 3).

The PHQ-9 score decreased in both treatment groups by week 6 by a similar extent, followed by an insignificant continued decline in scores in both treatment groups by week 12 (Fig. 2C and Table 3). Improvements in PHQ-9 scores in the first 6 weeks remained significant in both treatment groups after statistical adjustment, with a noted − 2.5 (SE, 1.1; *P* = 0.03) decline in incremental group and − 3.5 (SE, 1.5; *P* = 0.02) decline in conventional group (Table 3).

### Participant feedback

In aggregate, 86% of respondents rated their overall research experience highly, 90% reported good comprehension of study-related assessments, 97% felt their healthcare was not jeopardized, 72% affirmed being reminded to perform urine collection aided task completion, 74% indicated timed urine collections were not burdensome, and 83% expressed their motivation for study participation was to help advance the care of patients with KDRD.

## Discussion

This pilot study showed dynamic, longitudinal changes in patient-reported outcomes with a trend for differences noted between domains assessed and between treatment groups. Specifically, the DSI improved (i.e., scores decreased) over time in both treatment groups, with higher improvements seen with thrice-weekly HD. In contrast, symptoms of anxiety (GAD-7 score) and depression (PHQ-9 score) were better (i.e., lower scores) while on schedules of twice-weekly HD, with results slightly moderated after adjustment for baseline covariates.

Longitudinal changes in patient-reported symptomatology and satisfaction with their care in people afflicted with kidney disease has received little attention [27]. However, the importance of including patient-reported outcome measures that illustrate patient priorities for their healthcare has garnered growing recognition. Clinical trials are increasingly analyzing patient’s perspectives on treatment-related physical, functional and psychological impact [28]. Outcomes important for the patients, and characterized by the patients, have the potential to influence healthcare policy and thus change practice paradigms [29–31]. Thus, patient translation of treatment effects is becoming an essential tool to guide better allocation of funds and maximize the impact of the results for patients and society [32]. With regards to KDRD, the period of dialysis initiation is demarcated by an abrupt decline in individual’s independence and a surge in morbidity, with excess mortality befalling during the first 6–12 months of HD therapy [33]. One potential approach to mitigate some of the initial psychological burdens at dialysis initiation is to adopt the incremental approach, adding HD time or HD sessions as kidney residuals wane. To date, the effects of incrementally-prescribed twice-weekly HD and residual kidney function on health-related quality of life remain poorly delineated. Based on cross-sectional data from the China Dialysis Outcomes and Practice Patterns Study, patients dialyzing two times per week (26% of the patients in China) had longer treatment times and lower standardized Kt/V, but similar quality of life scores [18]. In a prospective cohort study of 734 patients with incident KDRD in the US, those with urine output at baseline (defined as producing at least 250 mL/day) reported overall better QOL (*P* = 0.05) and less dietary restriction (*P* = 0.05) [19]. Thus, longitudinal comparisons of patient-reported outcomes based on HD treatment schedule remains of particular interest. Thus, longitudinal comparisons of patient-reported outcomes based on HD treatment schedule remains of particular interest.

Notwithstanding the growing recognition of patient-reported symptoms and experiences in clinical research, the approach to interpreting patient-reported outcomes in chronically ill patients is challenging as different measures may assess varied aspects of patients’ experiences. This pilot study generated a few important observations. First, there could be a dichotomy in patient-reported experiences based on domains assessed. While items assessed with the DSI instrument indicated treatment-group independent improvement in the aggregate of physical and emotional symptoms, physiological well-being followed a treatment-dependent trend with lower burden of anxiety-related psychological symptoms being reported during the time period of twice-weekly HD. Based on these findings, we surmise that the timing of questionnaire administration should be an important consideration in the design of future, larger clinical trials. Prior studies pointed to multidimensional features of patient-reported quality of life measures that vary over time, emphasizing the need for serial assessments [9, 34, 35]. For a clinical trial of twice-weekly HD start vs thrice-weekly HD, eliciting patient-reported symptoms *after* conversion from twice-weekly to thrice-weekly HD will miss the opportunity to properly characterize patient-reported outcomes by different schedules of HD treatment. Given that transition from twice- to thrice-weekly HD is patient dependent and may occur at any time point during KDRD trajectory, frequent rather than distant time points of questionnaire administration ought to be part of the study design. As such, sensible selection of short, validated questionnaires for frequent instrument administration will be necessary to avoid respondent burden and minimize the risk of missing data. Second, results interpretation may prove to be problematic. In this pilot study, changes in DSI score were larger (i.e., better) with thrice-weekly HD, possibly because straightway introduction of thrice-weekly HD on a background of long-standing kidney dysfunction afforded swifter improvement in symptoms associated with uremia (e.g., appetite) than twice-weekly HD. In contrast, changes in psychological symptoms were better with twice-weekly HD. Moreover, a response shift phenomenon may occur among patients treated with thrice-weekly HD wherein patients experience a change in the meaning of their quality of life as a form of coping with illness. In such instance, patient-reported scores may improve not necessarily because quality of life per se has improved, diminishing the chance of finding differences between treatment groups even when differences may exist [36]. Future studies should encompass the *then-test* to assess whether, how and to what extent response shift occurred [37]. Statistical analysis aside, what symptom domain matters more to patients in general and, more importantly, to each individual and at different stages in a patient’s disease trajectory should remain at the core of deciding dialysis prescription. Therefore, while patient-reported instruments are useful tools for clinical investigators, providers ought to continue to adapt clinical care based on each individual’s needs. Last, feedback obtained from participants helped the investigators understand what study-related processes of care require refinement. For example, the pilot study entailed inter-dialysis urine collection during follow-up, with timeframes of urine collection ranging from 45 to 68 hours. Employing equations of calculating residual kidney function based on 24-hour urine collection will likely decrease procedure burden on the patients [38]. Frequent interaction with patients and reminder to perform urine collection were reported to help with task completion, making this strategy an important and simple tool to increase participant adherence to study protocol.

Results ought to be discussed through the lens of what constitutes a minimal important change, which is the smallest change in score in the construct measured that patients perceive as important [39]. Importantly, the minimal important change is not a fixed characteristic of any patient-reported outcome measure; instead, it can vary across populations, disease severity, settings, study designs and analyses used to estimate this metric. Prior work suggested a minimum change in DSI score of 7 points [40], in GAD-7 score of 4 points [41], and in PHQ-9 score of 5 points [42] as the cut-offs for minimal important change. Nevertheless, this metric has not been expressively elicited in patients treated with incremental HD. Therefore, with future studies, it will be important to establish a minimal important change anchored in the experiences and perceptions of patients treated with incremental HD.

Our study has limitations. Our study was not powered to detect statistical differences in patient-reported outcomes, but rather to explore the feasibility of implementing our trial procedures and to inform a power calculation for a future randomized controlled trial [22]; thus, the results ought to be interpreted with caution. Due to the nature of the intervention, patients and providers were not blinded to allocated treatments, thereby weakening construct validity. The study was conducted at a single health system organization, thus limiting its generalizability. Receipt of HD according to the conventional thrice-weekly HD prior to study enrollment may have influenced patient-reported outcomes, albeit the extent to which this occurred remains speculative. In our pilot study, transition from twice- to thrice-weekly HD was defined a priori while in real life, the timing of transition cannot be precisely foretold. Future and larger clinical trials of incremental HD will need to emulate real-life prescription of incremental HD. Future studies should also analyze longitudinal effects on psychological burdens related to kidney function-tailored HD frequency. It is conceivable that while an initial HD schedule of twice-weekly HD may subtract from the initial stress and burden associated with thrice-weekly HD, the trade-off may consist in a later episode of anxiety related to increasing HD frequency. A recent pilot study done in the UK reported no signal of benefit of incremental HD, compared to conventional HD, in terms of patient-reported outcomes measured with EuroQol 5D-5L, PHQ-9, Illness Intrusiveness Rating Scale and Montreal Cognitive Assessment measured at baseline and months 6 and 12 [43]. However, in the study done in the UK, patient-reported outcomes were collected at fixed time intervals and many of the patients originally treated with twice-weekly HD may had already experienced conversion to thrice-weekly HD at the time of questionnaire administration. Overall, the current studies cannot be interpreted as presence—or lack thereof—of a monotonic association between incremental HD and psychological symptoms until future larger clinical trials will include frequent measurements of patient-reported outcomes to detect if dynamic effects on psychological burdens exist.

In conclusion, this study suggests the prescribed HD treatment frequency may impact patient-reported psychological symptoms, with more HD dependency causing higher symptom burden of depression and anxiety. This approach, however, needs to be subjected to rigorous randomized clinical trials.

## Supplementary Information

**Additional file 1: Table S1.** Eligibility criteria.

**Additional file 2: Table S2.** Participant feedback questionnaire.

## Data Availability

The datasets used during the current study are available from the corresponding author on reasonable request.

## Abbreviations

KDRD: Kidney dysfunction requiring dialysis
HD: Hemodialysis
DSI: Dialysis Symptom Index
GAD-7: Generalized Anxiety Disorder 7 items
PHQ-9: Patient Health Questionnaire 9 items
