# Supplementary material for: Patient-reported outcomes in a pilot clinical trial of twice-weekly hemodialysis start with adjuvant pharmacotherapy and transition to thrice-weekly hemodialysis vs conventional hemodialysis
Source: BMC Nephrol. 2022 Sep 27;23:322. doi: 10.1186/s12882-022-02946-w (PMC9513956; doi:10.1186/s12882-022-02946-w)
Supplement: Supplementary file 1 — Additional file 1: Table S1. Eligibility criteria. [file 12882_2022_2946_MOESM1_ESM.docx]

| **Table S1. Eligibility criteria** |
| --- |
| **Inclusion criteria** |
| Age ≥18 years |
| Require long term maintenance HD as deemed by their treating nephrologist |
| Had eGFR ≥5mL/min/1.73m^2^ at the time of HD initiation |
| Received ≤6 HD sessions by the expected date of enrollment and randomization |
| Have urine output ≥500mL per 24-hour timed urine collection at the time of screening |
| **Exclusion criteria** |
| Abrupt decline in kidney function preceding HD initiation  (i.e., eGFR was ≥30 mL/min/1.73 m^2^ within 3 months prior to HD initiation) |
| Have severe systolic cardiac dysfunction with left ventricular ejection fraction <30% |
| Active diagnosis of hepatorenal syndrome |
| Have a malignancy that is likely to impact survival |
| Have a medical condition that would jeopardize the safety of the subject |
| History of noncompliance that would jeopardize patient adherence to study protocol |
| Inadequate written and verbal English comprehension |
| Abbreviations: HD, hemodialysis; eGFR, estimated glomerular filtration rate assessed by the Chronic Kidney Disease Epidemiology Collaboration (CKD-EPI) equation |
