# Supplementary material for: Patient-reported outcomes in a pilot clinical trial of twice-weekly hemodialysis start with adjuvant pharmacotherapy and transition to thrice-weekly hemodialysis vs conventional hemodialysis
Source: BMC Nephrol. 2022 Sep 27;23:322. doi: 10.1186/s12882-022-02946-w (PMC9513956; doi:10.1186/s12882-022-02946-w)
Supplement: Supplementary file 2 — Additional file 2: Table S2. Participant feedback questionnaire. [file 12882_2022_2946_MOESM2_ESM.docx]

| **Table S2. Participant feedback questionnaire** | | | | | |
| --- | --- | --- | --- | --- | --- |
| **Dimensions and individual items within each dimension** | Use the Scale to Rate the Answers | | | | |
| **Information and communication** | | | | | |
| Overall study explained to my full understanding | Definitely Yes | Probably Yes | Probably No | Definitely No | I don’t know |
| Risks/benefits of joining the study explained | Definitely Yes | Probably Yes | Probably No | Definitely No | I don’t know |
| Study details were included in the informed consent docs | Definitely Yes | Probably Yes | Probably No | Definitely No | I don’t know |
| Informed consent document was understandable | Definitely Yes | Probably Yes | Probably No | Definitely No | I don’t know |
| **Coordination of care** | | | | | |
| Something happened that I was not well prepared for, which was related to the study | Definitely Yes | Probably Yes | Probably No | Definitely No | I don’t know |
| Understood which tests were for research  *[ask the patient to ensure he/she understood urine collections were for research]* | Definitely Yes | Probably Yes | Probably No | Definitely No | I don’t know |
| Did you feel that your healthcare was compromised during the study? | Definitely Yes | Probably Yes | Probably No | Definitely No | I don’t know |
| Do you think the patients need to be reminded every day about collecting the urine, during the period of urine collection, if the collection is longer than 1 day? | Definitely Yes | Probably Yes | Probably No | Definitely No | I don’t know |
| **Perception on study-related assessments: urine collections** | | | | | |
| During the study, did you feel that the urine collections were burdensome? | Definitely Yes | Probably Yes | Probably No | Definitely No | I don’t know |
| What do you think is the chance that a person might accidentally flush (or forget to collect) some of the urine voids when they need to collect the urine? | Very high | High | Low | Very Low | I don’t know |
| Do you think the patients will disclose if they brought an incomplete urine collection? | Definitely Yes | Probably Yes | Probably No | Definitely No | I don’t know |
| Do you think that collecting the urine every 2-3 months, for 1 or 2 years, would be manageable for most people on dialysis? | Definitely Yes | Probably Yes | Probably No | Definitely No | I don’t know |
| **Motivation** | | | | | |
| Why did you choose to participate in this study? |  |  |  |  |  |
| To contribute important information to medical science | Definitely Yes | Probably Yes | Probably No | Definitely No | I don’t know |
| To potentially help other people with similar conditions | Definitely Yes | Probably Yes | Probably No | Definitely No | I don’t know |
| I hoped that the research study would improve my medical condition | Definitely Yes | Probably Yes | Probably No | Definitely No | I don’t know |
| To gain insights into my own health | Definitely Yes | Probably Yes | Probably No | Definitely No | I don’t know |
| To benefit from the additional medical attention and testing that the study provided | Definitely Yes | Probably Yes | Probably No | Definitely No | I don’t know |
| Because of the financial incentives of the study | Definitely Yes | Probably Yes | Probably No | Definitely No | I don’t know |
